# Reversed argininosuccinate lyase activity in fumarate hydratase-deficient cancer cells

**DOI:** 10.1186/2049-3002-1-12

**Published:** 2013-03-21

**Authors:** Liang Zheng, Elaine D MacKenzie, Saadia A Karim, Ann Hedley, Karen Blyth, Gabriela Kalna, David G Watson, Peter Szlosarek, Christian Frezza, Eyal Gottlieb

**Affiliations:** 1 Cancer Research UK, Beatson Institute for Cancer Research, Switchback Road, Glasgow, G61 1BD, UK; 2 Strathclyde Institute of Pharmacy and Biomedical Sciences, University of Strathclyde, 27 Taylor Street, G4 0NR, Glasgow, UK; 3 Queen Mary, University of London, Barts and The London School of Medicine, Charterhouse Square, London, EC1M 6BQ, UK; 4 Department of Medical Oncology, St Bartholomew’s Hospital, West Smithfield, London, EC1A 7BE, UK; 5 Medical Research Council Cancer Cell Unit, Hutchison/MRC Research Centre, Hills Road, Cambridge, CB2 OXZ, UK

**Keywords:** HLRCC, Renal cancer, Fumarate hydratase, Argininosuccinate, Metabolomics

## Abstract

**Background:**

Loss of function of fumarate hydratase (FH), the mitochondrial tumor suppressor and tricarboxylic acid (TCA) cycle enzyme, is associated with a highly malignant form of papillary and collecting duct renal cell cancer. The accumulation of fumarate in these cells has been linked to the tumorigenic process. However, little is known about the overall effects of the loss of FH on cellular metabolism.

**Methods:**

We performed comprehensive metabolomic analyses of urine from Fh1*-*deficient mice and stable isotopologue tracing from human and mouse FH-deficient cell lines to investigate the biochemical signature of the loss of FH.

**Results:**

The metabolomics analysis revealed that the urea cycle metabolite argininosuccinate is a common metabolic biomarker of FH deficiency. Argininosuccinate was found to be produced from arginine and fumarate by the reverse activity of the urea cycle enzyme argininosuccinate lyase (ASL), making these cells auxotrophic for arginine. Depleting arginine from the growth media by the addition of pegylated arginine deiminase (ADI-PEG 20) decreased the production of argininosuccinate in FH-deficient cells and reduced cell survival and proliferation.

**Conclusions:**

These results unravel a previously unidentified correlation between fumarate accumulation and the urea cycle enzyme ASL in FH-deficient cells. The finding that FH-deficient cells become auxotrophic for arginine opens a new therapeutic perspective for the cure of hereditary leiomyomatosis and renal cell cancer (HLRCC).

## Background

Germline mutations in the tricarboxylic acid (TCA) cycle enzyme fumarate hydratase (FH, EC 4.2.1.2) lead to hereditary leiomyomatosis and renal cell cancer (HLRCC, OMIM #150800), a genetic cancer syndrome characterized by benign smooth muscle tumors, uterine fibroids, and a highly malignant form of papillary and collecting duct renal cell cancer. HLRCC patients carry heterozygous germline mutations of *FH*, but loss of heterozygosity occurs in the tumors leading to a complete loss of FH [1]. The tumorigenic process of HLRCC is characterized by early onset of renal cysts that progress into malignant carcinoma [2]. Furthermore, reduction of FH expression is common in renal cancers without *FH* germline mutations [3]. Mice with kidney-specific biallelic deletion of *Fh1* develop severe renal cysts. However, these cysts do not progress into renal carcinoma [4].

It is not yet clear how the loss of FH predisposes patients to renal cancer. The accumulation of fumarate was identified as one of the most striking biochemical features of FH-deficient cells, with a potential tumorigenic role [5,6]. Indeed, it was initially thought that the stabilization of the transcription factor hypoxia-inducible factor (HIF), caused by the accumulation of fumarate, played a key role in the tumorigenesis of FH-deficient renal cancer [5-7]. However, the relevance of HIF as a tumor driver has been recently challenged by the observation that the genetic ablation of HIF in Fh1-deficient mice did not abolish cyst formation but, on the contrary, exacerbated this phenotype [8]. A novel link between fumarate accumulation and tumorigenesis was later proposed; fumarate was found to covalently modify cysteine residues of Keap1, the negative regulator of the transcription factor Nrf2, suggesting a role for a deregulated antioxidant response in the formation of FH-deficient tumors [8,9].

While the mechanisms of tumorigenesis in FH-deficient cells have been extensively investigated, the metabolic changes caused by the loss of FH activity have only been partially addressed. By using Fh1-deficient mouse epithelial kidney cells we have recently shown that in the absence of Fh1, the TCA cycle is truncated causing an accumulation of fumarate and succinate paralleled by a decrease of malate and citrate. The accumulation of TCA cycle metabolites in *Fh1*^*−/−*^ cells diverts TCA metabolites into heme biosynthesis and degradation, engaging a linear metabolic pathway beginning with glutamine uptake and ending with bilirubin excretion [10]. Of note, one of the key enzymes of this pathway, heme oxygenase-1, is an Nrf2 target gene, underscoring the role of an activated antioxidant response in the survival of FH-deficient cells [11]. An additional metabolic adaptation was identified in human FH-deficient cells. It was demonstrated that, by reversing parts of the TCA cycle, the reductive carboxylation of alpha-ketoglutarate (AKG) to isocitrate can generate citrate and malate from glutamine, thus providing anabolic building blocks required for cell proliferation [12].

The loss of FH is expected to have broader effects on cellular metabolism. Metabolomic analyses are now essential tools to investigate cell metabolism which have been recently exploited to identify cancer-specific metabolic pathways, such as serine metabolism [13,14] and reductive carboxylation of glutamine-derived AKG [12,15]. Further to providing important insights into cellular metabolism, metabolomic investigation of body fluids, such as urine and blood, may be an invaluable tool for finding novel cancer-specific metabolic biomarkers. In this work, we performed metabolomic investigations of urine from Fh1*-*deficient mice and of growth media of FH-deficient cell lines. We identified a distinct metabolic signature, marked by the abundant secretion of fumarate and the urea cycle metabolite argininosuccinate. Furthermore, we demonstrated that argininosuccinate is produced from arginine and fumarate by the reversed activity of the urea cycle enzyme argininosuccinate lyase (ASL, EC 4.3.2.1). Finally, by using an arginine-depleting enzyme with US Food and Drug Administration (FDA) orphan drug status, pegylated arginine deiminase (ADI-PEG 20) [16], we demonstrated that blocking the conversion of arginine to argininosuccinate specifically reduces cell proliferation of FH-deficient cells, opening new opportunities for the treatment of FH-deficient renal cell cancer.

## Methods

### Animal work

All work was performed in accordance with the Animals (Scientific Procedures) Act 1986.

### Cell culture

*Fh1*^*fl/fl*^, *Fh1*^*−/−*^*,* UOK262 and UOKpFH cell lines were obtained and cultured as previously described [10]. In brief, all cell lines were cultured in DMEM supplemented with 10% FBS and 2 mM glutamine. The mouse cell line was additionally supplemented with 1 mM pyruvate and 50 μg/mL uridine.

### Ultrasound

High resolution ultrasound imaging of mouse kidneys was performed as previously described [17], using a Vevo 770 system (Visual Sonics, Toronto, Canada) with a 25 MHz transducer and 6 mm depth scanhead. All procedures were carried out according to UK Home Office regulations.

### Quantification of renal cysts

Kidneys were removed from control mice at 12 months of age, fixed in 10% neutral buffered formalin and bisected before processing and paraffin embedding. Six 4 μm sections (taken at a 100 μm intervals) were cut through each kidney and stained with hematoxylin and eosin. Renal cysts were identified by the presence of a cuboidal epithelial lining and those with a diameter above 50 μm were scored as positive. The average number of cysts per section for each animal was calculated.

### Immunohistochemistry

Formalin fixed, paraffin embedded sections were dewaxed and rehydrated before antigen retrieval by microwaving in citrate buffer pH 6. Sections were incubated overnight at 4°C with rabbit anti-fumarase (Autogen Bioclear, Calne, UK) and stained using the EnVision kit (Dako, Glostrup, Denmark), according to the manufacturer’s instructions.

### Detection of Cre-mediated recombination

Ten μm cryosections were cut from snap frozen kidneys and β-galactosidase activity was measured using X-gal substrate following a standard procedure (IHC World, Woodstock, MD, USA).

### Metabolomic extraction of mouse urine

Urine was collected and immediately processed for deproteinization by dilution 1:3 with water and then 1:3 with acetonitrile (by volume). The suspension was then vortexed and immediately centrifuged at 16,000 g for 15 minutes at 0°C. The transparent supernatant was then submitted to liquid chromatography-mass spectrometry (LC-MS) metabolomic analysis.

### Metabolomic extraction of cells

A total of 5 × 10^5^ cells were plated onto 6-well plates and cultured in standard medium for 24 hours. For the intracellular metabolomic analysis, cells were quickly washed three times with PBS to remove contaminations from the metabolites in the media. The PBS was aspirated and cells were lysed by adding a pre-cooled extraction solution (ES) composed of 50% methanol and 30% acetonitrile in water. Cell number was counted in a parallel control dish, and cells were lysed in 1 ml of ES per 2 × 10^6^ cells. The lysates were vortexed for 5 minutes at 4°C and immediately centrifuged at 16,000 g for 15 minutes at 0°C. The supernatants were collected and analyzed by LC-MS. For the metabolomic extraction of spent media, the media was diluted 1:3 with water and then deproteinized 1:3 with acetonitrile. The supernatant was then processed as described above. Fresh medium without cells was incubated in the same experimental conditions and used as a reference.

### Metabolomic extraction of mouse kidneys

Ten mg of freshly excised mouse kidneys were lysed in 250 μL of a 50% methanol and 30% acetonitrile aqueous solution using Precellys 24 lysing tubes (tissue homogenizing CKMix - KT03961-1-009.2; Bertin Technologies, Montigny-le-Bretonneux, France), following the manufacturer’s instructions. The tissue lysate was immediately centrifuged at 16,000 g for 15 minutes at 0°C. The supernatant was collected and analyzed by LC-MS.

### LC-MS metabolomic analysis

For the LC separation, column A was the ZIC-HILIC (150 mm × 2.1 mm, internal diameter (id) 5 μm; SeQuant, Umeå, Sweden) with a guard column (20 mm × 2.1 mm id 5 μm; Hichrom, Reading, UK). Mobile phase A: 0.1% formic acid v/v in water. Mobile phase B: 0.1% formic acid v/v in acetonitrile. The flow rate was kept at 100 μL/min and gradient was as follows: 0 minutes 80% of B, 12 minutes 50% of B, 26 minutes 50% of B, 28 minutes 20% of B, 36 minutes 20% of B and 37 to 45 minutes 80% of B. Column B was the ZIC-pHILIC (150 mm × 2.1 mm id 5 μm; SeQuant) with the guard column (20 mm × 2.1 mm id 5 μm; Hichrom). Mobile phase C: 20 mM ammonia carbonate plus 0.1% ammonia hydroxide in water. Mobile phase D: acetonitrile. The flow rate was kept at 100 μL/min and gradient as follows: 0 minutes 80% of D, 30 minutes 20% of D, 31 minutes 80% of D and 45 minutes 80% of D. The Exactive Orbitrap mass spectrometer (Thermo Scientific, Waltham, MA, USA) was operated in a polarity switching mode.

### Quantification of metabolites

Standard compounds were dissolved in water (concentration range of each metabolite between 1 mM and 10 mM) and then further diluted in a 50:50 acetonitrile:water solution (solution B, concentration between 20 μM and 200 μM). Cells or media extracts (200 μl) were then mixed with 800 μl of dilution solvent, containing 0, 4, 20, 100, 300 or 500 μl of stock solution B. Samples prepared as indicated were analyzed by LC-MS. The concentration of each metabolite in the extract was calculated according to a linear regression fit. All dilution series were performed in triplicate using three biological replicates.

### ^13^C-glutamine and arginine labeling experiments

A total of 5 × 10^5^ cells were plated onto 6-well plates and cultured in standard medium for 12 hours. The medium was then replaced by fresh medium supplemented with either 2 mM U-^13^C-glutamine or 0.4 mM U-^13^C-arginine for the indicated time.

### Bioinformatics processing and statistical analysis of the metabolomic data

The cells and media data were log2 transformed and the replicates normalized using quantile normalization. A multi-factor analysis of variance and multiple test correction were then used to identify metabolites significantly different between experimental groups. Analyses were performed using Partek Genomics Suite software (version 6.5 and R version 2.14.0; Partek, St Louis, MO, USA).

### Short hairpin RNA (shRNA) interference of human and murine *ASL*

The lentiviral shRNA plasmids against human (NM_000048) and murine (NM_133768.4) *ASL* and the non-targeting control (RHS 4346) were purchased from Open Biosystems (Thermo Scientific). The shRNA for human cells was identified as: V3LHS_375513. The murine shRNA were: V3LMM_425165 and V3LMM_425167. All the sequences are free to download from the website: http://www.thermoscientificbio.com/openbiosystems.

The viral supernatant was obtained from the filtered growth media of the packaging cells HEK293T transfected with appropriate packaging plasmids and the relevant shRNA pGIPZ plasmids using calcium phosphate procedure. Next 2 × 10^5^*Fh1*^−/−^ and UOK262 cells were plated onto 6-well plates and infected with the viral supernatant in the presence of 4 μg/mL polybrene. After 2 days, the medium was replaced with selection medium containing 4 μg/mL puromycin. Infection efficiency and shRNA expression were tracked by monitoring the expression levels of TurboGFP, a fluorescent protein expressed by the bicistronic pGIPZ vector.

### Determination of expression levels of *ASL*

mRNA was extracted as previously described [10]. One μg of mRNA was retro-transcribed into cDNA using the High Capacity RNA-to-cDNA Kit (Life Technologies, Carlsbad, CA, USA). For the qPCR reactions, a duplex system of FAM-labeled primers for *ASL* (Hs00902699_m1 and Mm01197741_m1), VIC-labeled actin (Mm01205647_g1 for mouse) and lamin (Hs01059202_m1 for human) was used. One μL of TaqMan Fast Gene Expression Master Mix (Life Technologies); 1 μL of each primers and probes; and 4 μL of a 1:10 dilution of cDNA in a final volume of 20 μL were used. The primers were designed using the Applied Biosystems website. qPCR was performed in the 7500 Fast Real-Time PCR System (Life Technologies) using the TaqMan Fast program and expression levels of the indicated genes were calculated using the ΔΔC_t_ method by the appropriate function of the software using actin or lamin as calibrators.

### Treatment with ADI-PEG 20

A total of 1 × 10^5^ cells were plated onto 24-well plates and counted every 24 hours for 6 consecutive days using an Innovatis CASY cell counter (Roche, Hague Road, IN, USA). For arginine deprivation experiments, ADI-PEG 20 (Polaris Group, San Diego, CA, USA) was dissolved in cell culture media at the indicated concentration and replenished every 2 days. Clonogenic survival assay was performed as previously described [10], and 100 *Fh1*^*fl/fl*^ and 600 *Fh1*^*−/−*^ cells were plated per well in a 6-well plate. Twenty-four hours after plating, ADI-PEG 20 was added to the medium. Every 2 days, for a total of 8 days, fresh ADI-PEG 20 was added to the culture media. At the end of the treatment, the medium was replaced with fresh medium without the drug/vehicle and cells were left to grow for a further 4 days. The cells were then fixed by adding ice-cold trichloroacetic acid onto the plate at the final concentration of 3% for 1 hour at 4°C. Cells were then washed with water and air-dried. Then, cells were stained using 0.47% sulforhodamine B (SRB) for 30 minutes at room temperature. SRB was removed and cells were washed with 1% acetic acid and air-dried. Plates were scanned at the resolution of 600 dpi, 24-bit and saved as TIFF files. Each experiment was performed in triplicate plates.

## Results

**Figure 1:**
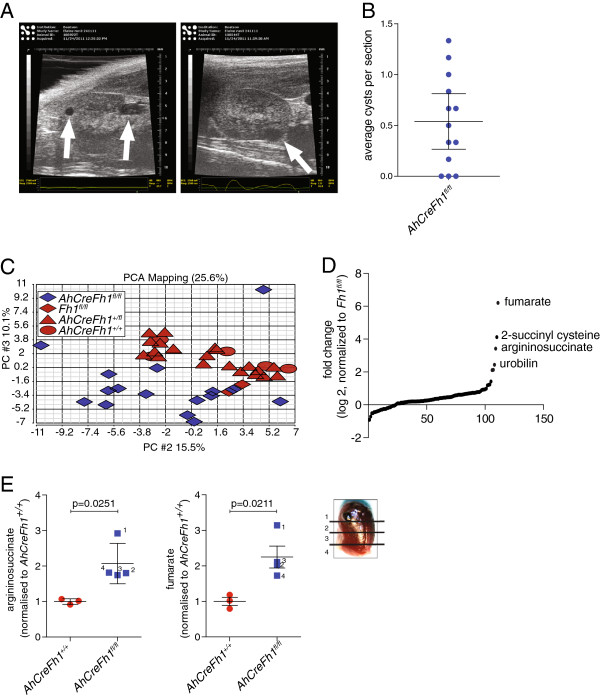
**Identification of urine biomarkers in *AhCreFh1***^***fl/fl ***^**mice. (A)** Representative images from the ultrasound scanning of the kidneys of *AhCreFh1*^*fl/fl*^ mice. Arrows indicate the cysts. **(B)** Quantification of cyst number in kidney sections from 1-year-old *AhCreFh1*^*fl/fl*^ mice performed by manual counting. Cysts with an average diameter above 50 μm were counted. **(C)** Principal component analysis (PCA) of the LC-MS urine metabolomic data obtained from mice with the indicated genotype. **(D)** Sigmoidal plot representation of the ratio of urinary metabolites based on the comparison between *AhCreFh1*^*fl/fl*^ and *Fh1*^*fl/fl*^ mice. Metabolites of interest are indicated. **(E)** LC-MS analyses of argininosuccinate and fumarate in kidney samples of *AhCreFh1*^*fl/fl*^ and *Fh1*^*fl/fl*^ mice sectioned as indicated in the figure. LC-MS, liquid chromatography-mass spectrometry; PCA, principal component analysis.

In order to study the metabolic alterations and adaptations of FH-deficient tumors *in vivo* we used a previously described mouse model with a conditional *Fh1* gene deletion, *Fh1*^*fl/fl*^[4]. These mice were crossed to transgenic mice expressing the Cre recombinase under the control of a beta-napthoflavone-inducible promoter Cyp1A (*AhCre*) and a lox-stop-lox lacZ reporter cassette within the Rosa26 locus [18] (Additional file 1A). In the absence of beta-napthoflavone, the *AhCre* mice stochastically express the Cre transgene in cells within multiple organs, such as the kidney, liver, heart and bladder [19,20]. Consistent with this notion, random recombination events in the absence of the inducer, with consequent generation of Fh1-deficient cells, were detected by tail genotyping and by lacZ expression in kidneys of *AhCre* and *LacZ* positive *Fh1*^*fl/fl*^ mice, indicated thereafter as *AhCreFh1*^*fl/fl*^ (Additional file 1B and C). Importantly, the stochastic loss of Fh1 in these mice resulted in the early formation of renal cysts, as identified by ultrasound scanning of *AhCreFh1*^*fl/fl*^ mice from the age of 6 months (Figure 1A). *Post mortem* analyses of 1-year-old *AhCreFh1*^*fl/fl*^ mice revealed the presence of multiple macroscopic renal cysts (Figure 1B and Additional file 1D). Immunohistological analysis of *AhCreFh1*^*fl/fl*^ kidneys confirmed the loss of Fh1 expression in the cystic regions (Additional file 1E).

It is noteworthy that, despite the likeliness of multiple stochastic events of gene deletion in these mice, the only observed hyperplasia was in the kidney. Together with the fact that FH loss-of-function is associated with renal cancer, these observations indicate that kidney cells may better survive the loss of the TCA cycle enzyme FH. Therefore, in order to investigate the metabolic signature associated with the loss of Fh1 in these mice, the urine of *AhCreFh1*^*fl/fl*^ mice and littermate controls were collected and analyzed by LC-MS. To increase the statistical robustness of this analysis, urines were collected on 3 separate days. The principal component analysis (PCA) of the metabolomic data revealed a clear separation between the *AhCreFh1*^*fl/fl*^ and the Fh1-proficient mice (Figure 1C). Importantly, the absence of metabolic sub-clustering between the *Fh1*^*+/+*^ and *AhCreFh1*^*fl/+*^ suggests that a monoallelic loss of Fh1 is not sufficient to generate a metabolic signature (Figure 1C), in line with the fact that no renal cysts were detected in *Fh1* heterozygous mice (not shown). The metabolites that consistently contributed to the urine metabolic signature of *AhCreFh1*^*fl/fl*^ mice were urobilin, S-(2-succinyl)cysteine (2SC), fumarate and argininosuccinate (Figure 1D). Considering the robustness of the metabolic signature, these metabolites may be considered biomarkers for HLRCC patients. Furthermore, the presence of urobilin (the urine form of bilirubin) in the urine of mice with Fh1-deficient cysts strongly supports our previous report that heme degradation is upregulated in FH-deficient tumors [10]. 2SC formation is likely due to cysteine addition to fumarate, as was previously reported to occur on proteins [8,21]. The presence of fumarate in the urine is not unexpected considering the high levels of fumarate detected in Fh1-deficient cells and tumors [4,5,10], but this is the first report which demonstrates the potential use of urine fumarate as a biomarker for early detection of renal cancer formation in HLRCC patients. The presence of high levels of argininosuccinate in the urine is somewhat surprising as this is a urea cycle metabolite. Nevertheless, like fumarate, argininosuccinate levels are higher in the Fh1-deficient cystic region of the kidney, indicating that the cysts are the source of these metabolites (Figure 1E).

**Figure 2:**
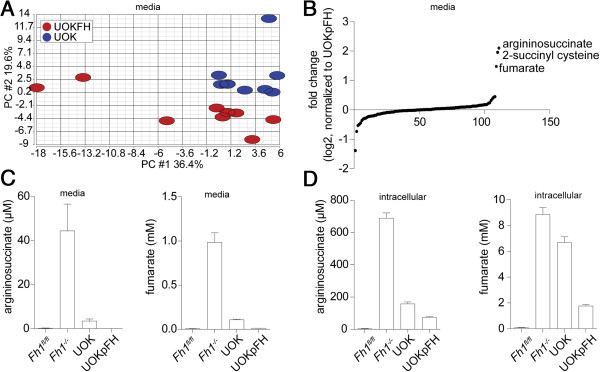
**Metabolic fingerprint of FH-deficient cell lines. (A)** PCA and **(B)** sigmoidal plot of the metabolomic data from growth media of the indicated cell lines. Results were obtained from nine independent cultures. UOK cells present a distinct metabolic signature compared to their FH-proficient counterpart, UOKpFH. **(C)** Extracellular and **(D)** intracellular fumarate and argininosuccinate levels in the indicated cell lines. Results were obtained from three independent cultures and represented as average ± SEM. PCA, principal component analysis; SEM, standard error of the mean; UOK, UOK262.

In order to test whether this could be extended to HLRCC, the metabolic profile of spent media of the patient-derived FH-deficient renal cancer cell line UOK262 [22], indicated hereafter as UOK, was compared to FH-reconstituted UOK cells (UOKpFH) [10]. The PCA revealed a distinct metabolic signature of UOK cells when compared to UOKpFH (Figure 2A). Importantly, in agreement with the metabolic signature of *AhCreFh1*^*fl/fl*^ mouse urine, 2SC, fumarate and argininosuccinate were the most increased metabolites (Figure 2B). Fumarate and argininosuccinate were also abundantly present in the media of the previously described [10]*Fh1*^*−/−*^ mouse epithelial kidney cells (Figure 2C) and also reached high intracellular levels in both human and mouse FH-deficient cell lines (Figure 2D).

Next we investigated the possible mechanisms of argininosuccinate accumulation in FH-deficient cells. Argininosuccinate is a urea cycle intermediate synthesized by argininosuccinate synthetase (ASS) from citrulline and aspartate, and subsequently converted to fumarate and arginine by ASL (Additional file 2A). Interestingly, argininosuccinate aciduria is observed in patients deficient in ASL. Therefore, it is plausible that an increase in aspartate- and citrulline-derived argininosuccinate is a consequence of ASL inhibition by high levels of fumarate in FH-deficient cells. However, cells were maintained in DMEM, which does not contain aspartate and no net uptake of the small amount of aspartate from the serum was detected by the described above extracellular metabolomic analysis (not shown). Therefore, the only source of aspartate in these cells is the TCA cycle (Additional file 2A). Since glutamine is the major carbon source of TCA cycle metabolites in these cells [10] (Additional file 3), efficient production of aspartate would normally require functional FH (Additional file 2A). Interestingly, however, it was recently demonstrated that glutamine-derived TCA cycle metabolites can be formed by reductive carboxylation of AKG in respiration-deficient cells [12,15]. Therefore, to test the possibility that aspartate and subsequently argininosuccinate are produced via this pathway in FH-deficient cells, we incubated cells with uniformly-labeled U-^13^C-glutamine and traced the heavy carbons (^13^C) incorporation into intracellular metabolites (isotopologues) by LC-MS (Additional file 2B, for a schematic representation of the labeling pattern).

**Figure 3:**
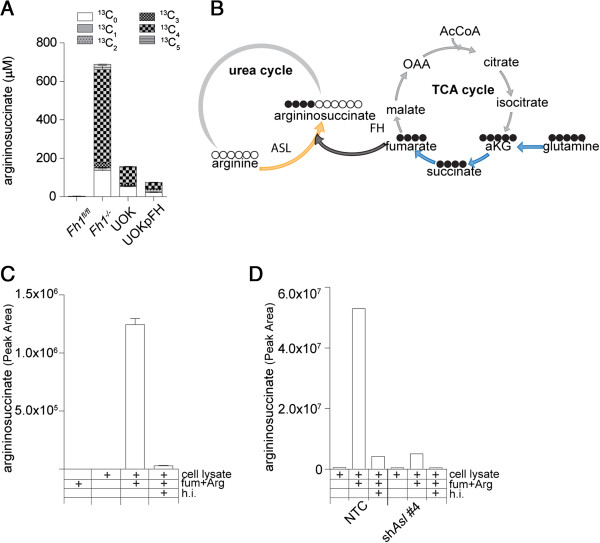
**Argininosuccinate is produced from arginine and fumarate. (A)** Isotopologue distribution analysis of intracellular metabolites after incubation with U-^13^C-glutamine for 24 hours. **(B)** In light of the isotopologues distribution (^13^C_4_) of glutamine-derived ^13^C-labeled argininosuccinate in FH-deficient cells (panel **A**), this scheme represents the most likely biochemical pathway that links TCA cycle metabolites in FH-deficient cells to the urea cycle and to argininosuccinate production. Arginine and fumarate were mixed in a physiological buffer in the presence or absence of protein lysates from (**C**) *Fh1*^*−/−*^ cells or (**D**) *Fh1*^*−/−*^ cells in which *ASL* expression was silenced. The formation of argininosuccinate is enzymatic and requires ASL. Where indicated, the cell extracts were heat inactivated (HI) prior to the *in vitro* reaction. Results were obtained from three independent experiments and expressed as average ± SEM. ASL, argininosuccinate lyase; FH, fumarate hydratase; HI, heat inactivated; SEM, standard error of the mean.

Unexpectedly, in both human and mouse FH-deficient cells the steady-state levels of aspartate were decreased, and more importantly aspartate was mostly unlabeled (Additional file 3). In sharp contrast, argininosuccinate was quickly labeled with glutamine-derived carbons (Additional file 3), but unlike the expected contribution of three ^13^C from glutamine via aspartate (Additional file 2B), argininosuccinate major isotopologue was labeled on four glutamine-derived ^13^C with a mass shift of four Daltons (^13^C_4_) (Figure 3A). This result suggests that argininosuccinate is not produced from citrulline and aspartate in FH-deficient cells but rather from arginine and fumarate (Figure 3B). This surprising observation may be a consequence of either enzymatic or non-enzymatic nucleophilic attack of arginine on the electrophilic double bond of fumarate (Michael addition). When fumarate and arginine were mixed together in physiological buffer, no formation of argininosuccinate was detected (Figure 3C). However, when dialyzed cell extracts from mouse kidney epithelia Fh1^−/−^ cells were added to the reaction, a substantial production of argininosuccinate was observed. This reaction was blocked when the protein extract was heat-inactivated (HI) (Figure 3C). These results suggest that argininosuccinate production *in vitro* is enzymatically catalyzed.

**Figure 4:**
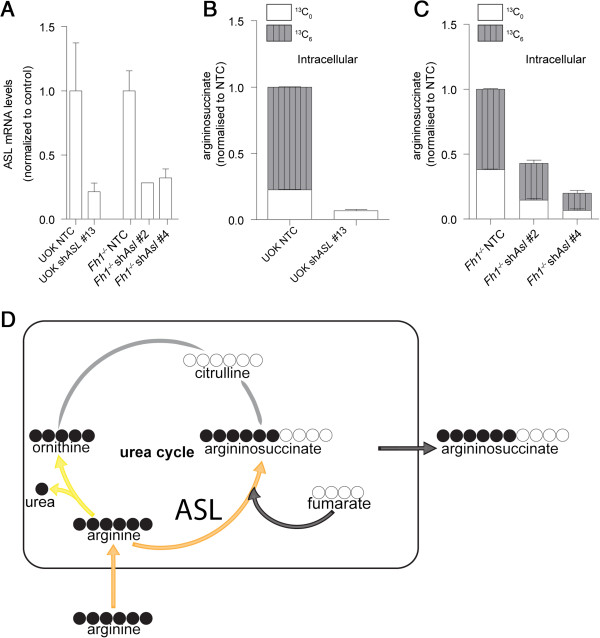
**Argininosuccinate is produced by the reverse activity of the urea cycle enzyme ASL. (A)** mRNA expression levels of *ASL* after acute infection of the indicated cell lines with shRNAs against human (sh*ASL*) or mouse (sh*Asl*) ASL. Values are normalized to cells infected with the control plasmid. Argininosuccinate production is decreased in *ASL*-silenced **(B)** human or **(C)** mouse cells. Cells were incubated with U-^13^C-arginine for 24 hours and the isotopologue distribution of argininosuccinate was analyzed by LC-MS. **(D)** Schematic representation of the fate of arginine in FH-deficient cells. Orange arrows indicate the major metabolic flux from arginine; light yellow arrow indicates low metabolic flux, gray arrows indicate undetectable metabolic flux; and dark gray arrow indicates the crosstalk between the TCA cycle and the urea cycle. All the results were obtained from three independent cultures and expressed as average ± SEM. ASL, argininosuccinate lyase; FH, fumarate hydratase; LC-MS, liquid chromatography-mass spectrometry; NTC, non-targeting control; SEM, standard error of the mean; shRNA, short hairpin RNA; TCA, tricarboxylic acid.

To test whether ASL is the enzyme required for the formation of argininosuccinate *in vitro*, cells were infected with a lentivirus encoding either short hairpin RNAs targeting *ASL* or non-targeting control shRNA (Figure 4A). When protein lysates from these cells were used as an enzymatic source for the *in vitro* reaction, it was clearly demonstrated that the silencing of *ASL* drastically reduced the *in vitro* production of argininosuccinate (Figure 3D). Overall, these results suggest that in the presence of excess fumarate, ASL is operating in reverse generating argininosuccinate from fumarate and arginine.

To validate this hypothesis, cells were cultured in the presence of U-^13^C-arginine and the isotopologue distribution of argininosuccinate was assessed. If the urea cycle is operating in the forward direction producing argininosuccinate from citrulline and aspartate, argininosuccinate would be labeled on five arginine-derived ^13^C (Additional file 4). However, if ASL is indeed working in reverse in these cells, argininosuccinate would be labeled on six arginine-derived ^13^C, due to the direct binding of unlabeled fumarate to labeled arginine (Additional file 4B). After 24 hours of incubation with U-^13^C-arginine, argininosuccinate was predominantly detected by mass-spectrometry as the ^13^C_6_ isotopologue (Figure 4B and C; NTC). In addition, only a very small amount of ^13^C-arginine-derived ^13^C_5_ ornithine and no ^13^C_5_ citrulline were detected (Additional file 4C). Finally, the silencing of *ASL* in FH-deficient cells (Figure 4A) caused a reduction in the steady-state levels of argininosuccinate, concomitant with a reduction in the production of the ^13^C_6_ argininosuccinate isotopologue from U-^13^C-arginine (Figure 4B and C; compare sh*Asl* to NTC). Altogether, these results clearly demonstrate that FH-deficient cells use exogenous arginine and intracellular fumarate to form argininosuccinate in a reversed ASL reaction (Figure 4D).

**Figure 5:**
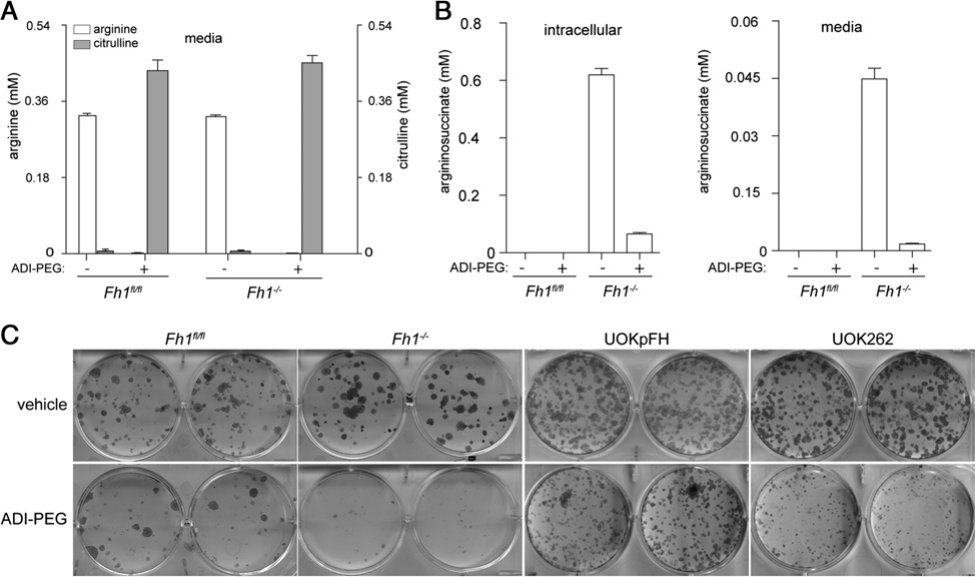
**Arginine depletion by ADI-PEG 20 reduces argininosuccinate and inhibits cellular proliferation in FH-deficient cells. (A)** Arginine levels determined in the media of the indicated cell lines after the incubation for 4 hours with 125 ng/mL ADI-PEG 20. **(B)** Excreted and intracellular levels of argininosuccinate after treatment with ADI-PEG 20, as in **(A)**, were detected by LC-MS. **(C)** Representative images of a colony survival assay after treatment with either 125 ng/mL (mouse cells) or 50 ng/mL (human cells) ADI-PEG 20 for 8 days. The media was replaced and cells were left to grow for a further 4 days before fixation and staining. All the results were obtained from three independent cultures and expressed as average ± SEM. FH, fumarate hydratase; LC-MS, liquid chromatography-mass spectrometry; SEM, standard error of the mean.

In order to further investigate the biological relevance of this novel biochemical adaptation mechanism, the biosynthesis of argininosuccinate was pharmacologically inhibited by depriving cells of arginine using ADI-PEG 20. This recombinant enzyme converts arginine into equimolar amounts of citrulline and ammonia. As expected, this enzyme rapidly converted medium arginine into citrulline (Figure 5A), caused a substantial drop of intracellular levels and secreted argininosuccinate in FH-deficient cells (Figure 5B). In order to investigate the effects of arginine deprivation, cells were cultured for 8 days with ADI-PEG 20 and a colony survival assay was performed. ADI-PEG 20 caused a measurable change in colony (cell) survival and it also affected colony size of both human and mouse FH-deficient cells (Figure 5C); indicating that these cells are auxotrophic for arginine, and that arginine deprivation affects both cell survival and proliferation.

## Discussion

The unexpected finding that mutations in the TCA cycle enzymes succinate dehydrogenase (SDH) [23] and FH [24], predispose to cancer, potently rekindled the field of cancer metabolism and promoted investigations of the link between aberrant metabolism and cancer formation. It is remarkable that, despite the fact that FH and SDH are part of the same biochemical pathway, their loss of function leads to different spectra of tumors [1]. In line with this, despite a multi-organ stochastic Fh1 loss in the *AhCreFh1*^*fl/fl*^ mice, the only observed phenotype was the development of renal cysts. We hypothesized that by excreting accumulated metabolites, kidney cells may better tolerate FH deficiency and would therefore enable fumarate to exert its onco-metabolic activity via the stabilization of the transcription factors HIF and Nrf2, and by promoting epigenetic changes via the inhibition of AKG-dependent dioxygenases [1].

Metabolomic analyses revealed that Fh1-deficient mice and cells accumulate and excrete significant amounts of fumarate and fumarate-related metabolites, among which, argininosuccinate predominated. Since argininosuccinate accumulation was consistently elevated in our studies, we propose that it may be a robust metabolic urine biomarker for the early detection of FH-associated renal cancer. Argininosuccinate is normally produced in the urea cycle from citrulline and aspartate, and is further converted into fumarate and arginine. Of note, this process is particularly important in the kidneys, since these organs help to maintain arginine homeostasis by producing arginine from dietary citrulline and releasing it to the blood stream [25]. In this work, we have shown that in the presence of excess fumarate in FH-deficient cells, this reaction is reverted and argininosuccinate is subsequently produced from fumarate and arginine. We have demonstrated that the urea cycle in these cells does operate in the forward direction to synthesize arginine and instead FH-deficient kidney cancers employ exogenous arginine to produce argininosuccinate. In addition, since ASL has been found recently to be required for systemic nitric oxide biosynthesis [26], the effects of fumarate on the urea cycle may extend to nitric oxide metabolism as well.

The secretion of fumarate appears to be the predominant strategy for FH-deficient cells to sustain viable, even though strikingly high, intracellular levels of fumarate. Nevertheless, we propose that ASL reversal partially contributes to fumarate detoxification and could have a role in the survival of FH-deficient cells. In fact, when cells were deprived of arginine using the recombinant enzyme, ADI-PEG 20, intracellular levels of argininosuccinate declined dramatically with severe consequences on cell proliferation. Although the genetic ablation of ASL has not been specifically investigated in the current study, it is tempting to speculate that other metabolic pathways might be exploited upon the loss of function of ASL to avoid fumarate accumulation.

## Conclusion

Our results highlight the complex and convoluted metabolic changes that occur after the loss of FH. In particular, these results suggest that FH-deficient cells have evolved mechanisms to compensate for the persistent intracellular accumulation of fumarate which would be otherwise toxic and would impair cellular proliferation. In addition, our approach provides a novel and practical way to screen for metabolic biomarkers in HLRCC and other FH-related kidney cancer patients. Finally, the observation that, unlike normal kidney epithelial cells, FH-deficient kidney cancer cells are auxotrophic for arginine provides potential new therapeutic interventions for these cancers.

## Abbreviations

2SC: S-(2-succinyl)cysteine; ADI-PEG 20: Pegylated arginine deiminase; AKG: Alpha-ketoglutarate; ASL: Argininosuccinate lyase; ASS: Argininosuccinate synthetase; DMEM: Dulbecco^′^s modified Eagle^′^s medium; ES: Extraction solution; FBS: Fetal bovine serum; FDA: Food and Drug Administration; FH: Human fumarate hydratase; Fh1: Murine fumarate hydratase; HI: Heat inactivated; HIF: Hypoxia-inducible factor; HLRCC: Hereditary leiomyomatosis and renal cell cancer; Id: Internal diameter; Keap1: Kelch-like ECH-associated protein 1; LC-MS: Liquid chromatography-mass spectrometry; Nrf2: Nuclear factor (erythroid-derived 2)-like 2; NTC: Non-targeting control; PBS: Phosphate buffered saline; PCA: Principal component analysis; qPCR: Real-time polymerase chain reaction; SDH: Succinate dehydrogenase; SEM: Standard error of the mean; shAsl: Short hairpin RNA targeting *Asl*; shRNA: Short hairpin RNA; SRB: Sulforhodamine B; TCA: Tricarboxylic acid.

## Competing interest

Authors declare no financial, personal, or professional interests that could be construed to have influenced their paper.

## Authors’ contribution

CF, EG and LZ conceived the study. CF and LZ performed the experiments with the help of EDM. EDM and SAK performed ultrasound experiments. KB supervised *in vivo* experiments and mating strategies for genetically modified animals. GK and AH analyzed and processed the metabolomics data. DGW assisted the metabolomics analyses. PS provided the ADI-PEG 20. CF and EG wrote the manuscript. All authors read and approved the final manuscript.

## Supplementary Material

Additional file 1 **Characterization of *AhCreFh1***^***fl/fl ***^**mice. (A)** Schematic representation of the *Fh1* allele in *Fh1*^*fl/fl*^ mice, and the construct used for the generation of *AhCre* and the *LacZ* reporter at the Rosa26 locus. **(B)** Tail clipping genotyping of *AhCreFh1*^*fl/fl*^ mice. The putative genotypes are indicated on the left, based on the expected size of the genomic PCR amplification products. *Fh1*^*+/+*^ = 230 bp, *Fh1*^*flfl*^ = 470 bp and *Fh1*^*-/-*^*=* 380 bp*.***(C)** Representative images of the lacZ staining performed on cryosections of dissected kidneys of the indicated mice. The bar indicates 200 μm. **(D)** Representative image (left) and relative hematoxylin and eosin staining (right) of a dissected kidney from *AhCreFh1*^*fl/fl*^ mice. The arrow on the left panel indicates a macrocyst. The bar on the right panel indicates 200 μm. **(E)** Immunohistochemistry for Fh1 performed on a kidney section of the indicated mouse strains. The bar indicates 200 μm. Click here for file

Additional file 2 **Schematic representation of the metabolic crosstalk between the urea cycle and the TCA cycle. (A)** In normal conditions, aspartate reacts with citrulline to produce argininosuccinate, a reaction catalyzed by ASS. Argininosuccinate is then converted into arginine and fumarate by ASL. **(B)** Schematic representation of a putative labeling of argininosuccinate in FH-deficient cells where the reductive carboxylation of AKG will generate labeled aspartate (^13^C_3_). Note that this representation does not take into account N-N chemical bound. AKG, alpha-ketoglutarate; ASL, argininosuccinate lyase; ASS, argininosuccinate synthetase; FH, fumarate hydratase; TCA, tricarboxylic acid. Click here for file

Additional file 3 **Isotopologue distribution of TCA cycle metabolites after incubation with U-**^**13**^**C-glutamine. (A)** Mouse and **(B)** human FH-deficient or proficient cell lines were incubated for the indicated time with 2 mmol/L U-^13^C-glutamine, and both the spent media and the intracellular metabolites were analyzed by LC-MS. The isotopologue composition is indicated in the legend and presented as peak area. FH, fumarate hydratase; LC-MS, liquid chromatography-mass spectrometry; TCA, tricarboxylic acid. Click here for file

Additional file 4 **Schematic representation of the hypothetic labeling profiles of argininosuccinate from U-**^**13**^**C-arginine-labelling experiments.** After the incubation with labeled arginine, argininosuccinate can be labeled either as **(A) **^13^C_5_ due to a fully functional urea cycle, or **(B)** as ^13^C_6_ if arginine is converted directly into argininosuccinate by the reversed activity of ASL. **(C)** Citrulline and ornithine detection by LC-MS in cells incubated with U-^13^C-arginine. ASL, argininosuccinate lyase; LC-MS, liquid chromatography-mass spectrometry. Click here for file
