# Supplementary figures and images for: Reversed argininosuccinate lyase activity in fumarate hydratase-deficient cancer cells
Source: Cancer Metab. 2013 Mar 21;1:12. doi: 10.1186/2049-3002-1-12 (PMC4108060; doi:10.1186/2049-3002-1-12)

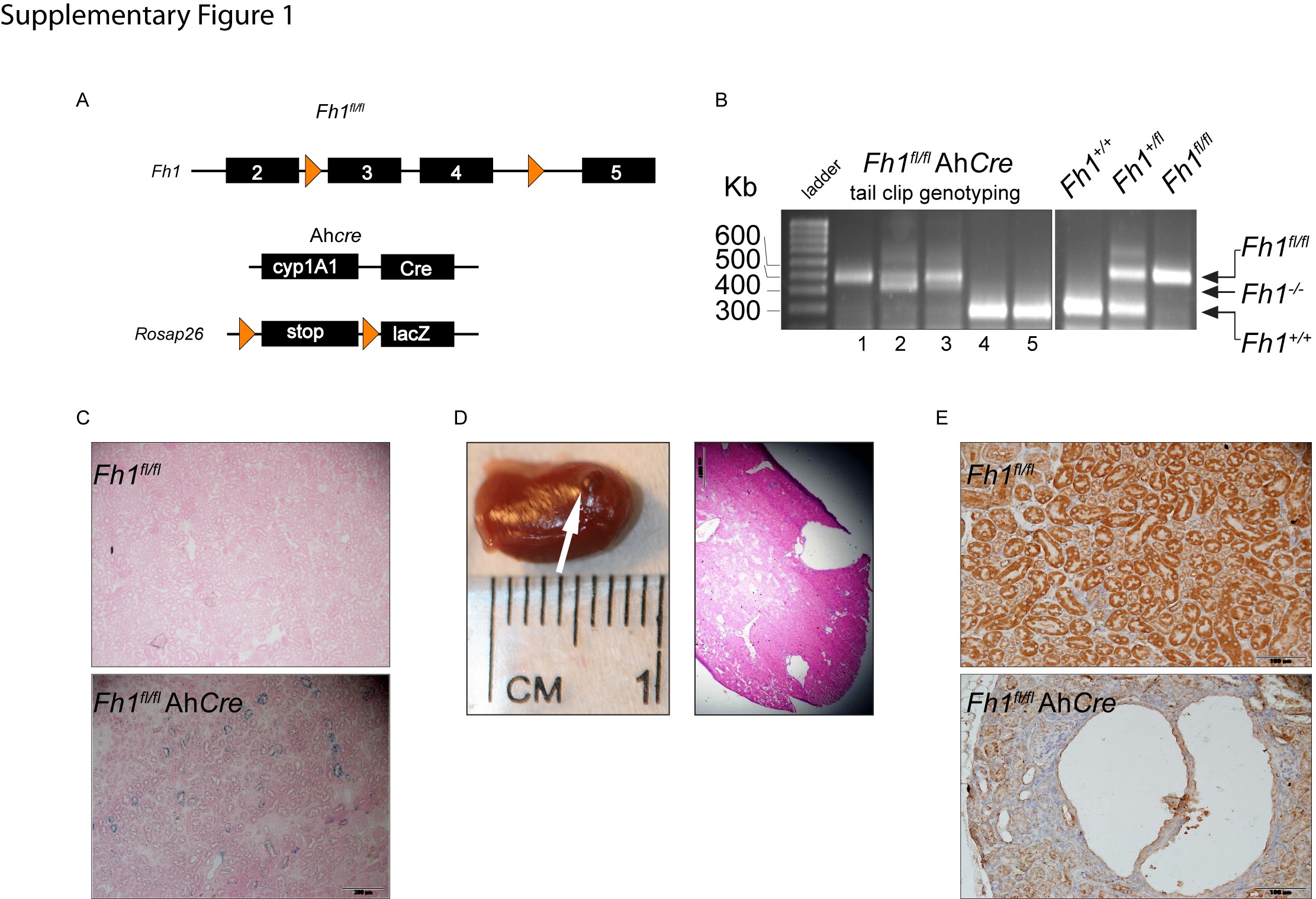

Supplement: Additional file 1 — Characterization of AhCreFh1 fl/fl mice. (A) Schematic representation of the Fh1 allele in Fh1 fl/fl mice, and the construct used for the generation of AhCre and the LacZ reporter at the Rosa26 locus. (B) Tail clipping genotyping of AhCreFh1 fl/fl mice. The putative genotypes are indicated on the left, based on the expected size of the genomic PCR amplification products. Fh1 +/+ = 230 bp, Fh1 flfl = 470 bp and Fh1 -/- = 380 bp.(C) Representative images of the lacZ staining performed on cryosections of dissected kidneys of the indicated mice. The bar indicates 200 μm. (D) Representative image (left) and relative hematoxylin and eosin staining (right) of a dissected kidney from AhCreFh1 fl/fl mice. The arrow on the left panel indicates a macrocyst. The bar on the right panel indicates 200 μm. (E) Immunohistochemistry for Fh1 performed on a kidney section of the indicated mouse strains. The bar indicates 200 μm. [file 2049-3002-1-12-S1.tiff]

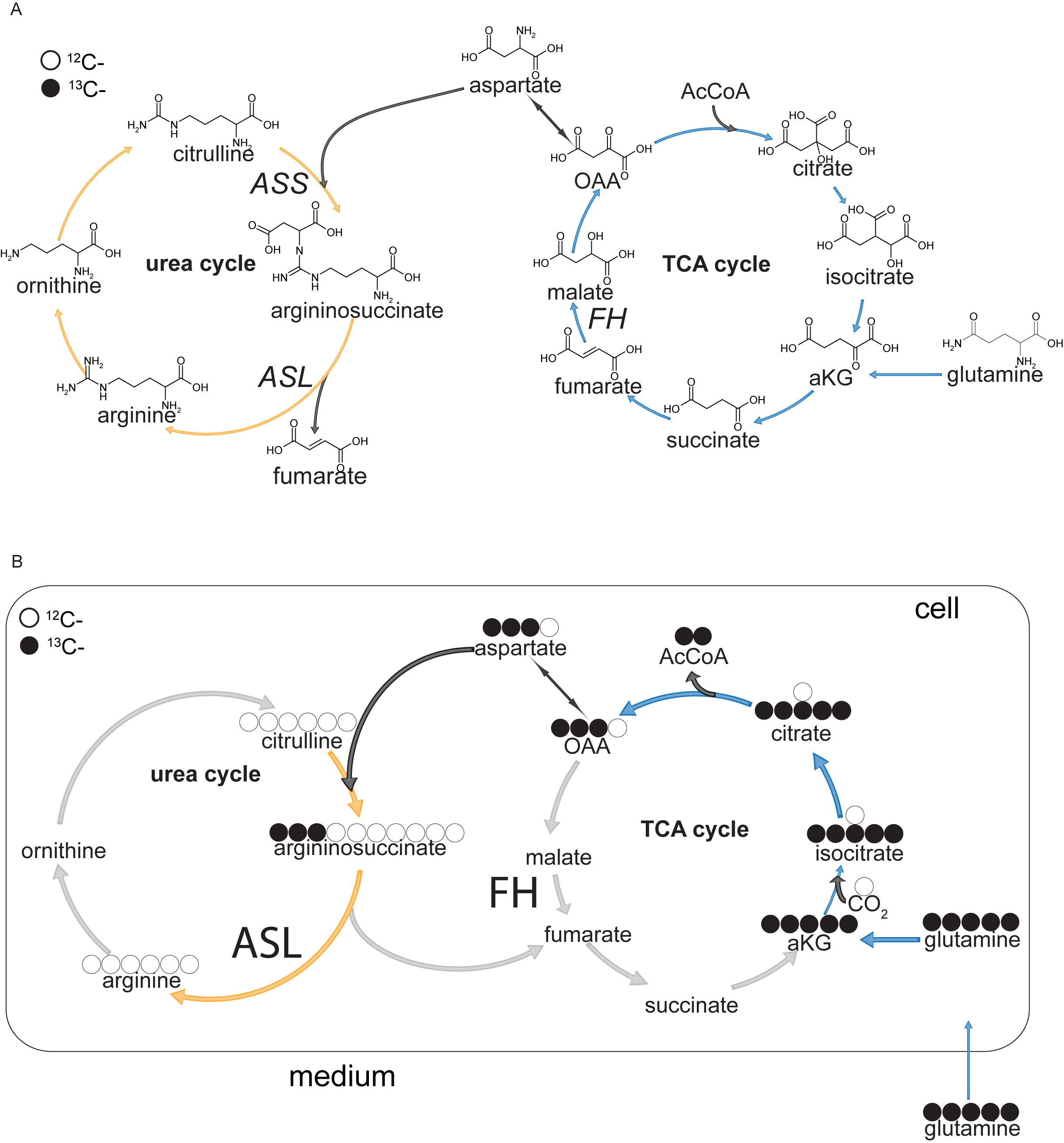

Supplement: Additional file 2 — Schematic representation of the metabolic crosstalk between the urea cycle and the TCA cycle. (A) In normal conditions, aspartate reacts with citrulline to produce argininosuccinate, a reaction catalyzed by ASS. Argininosuccinate is then converted into arginine and fumarate by ASL. (B) Schematic representation of a putative labeling of argininosuccinate in FH-deficient cells where the reductive carboxylation of AKG will generate labeled aspartate (13C3). Note that this representation does not take into account N-N chemical bound. AKG, alpha-ketoglutarate; ASL, argininosuccinate lyase; ASS, argininosuccinate synthetase; FH, fumarate hydratase; TCA, tricarboxylic acid. [file 2049-3002-1-12-S2.tiff]

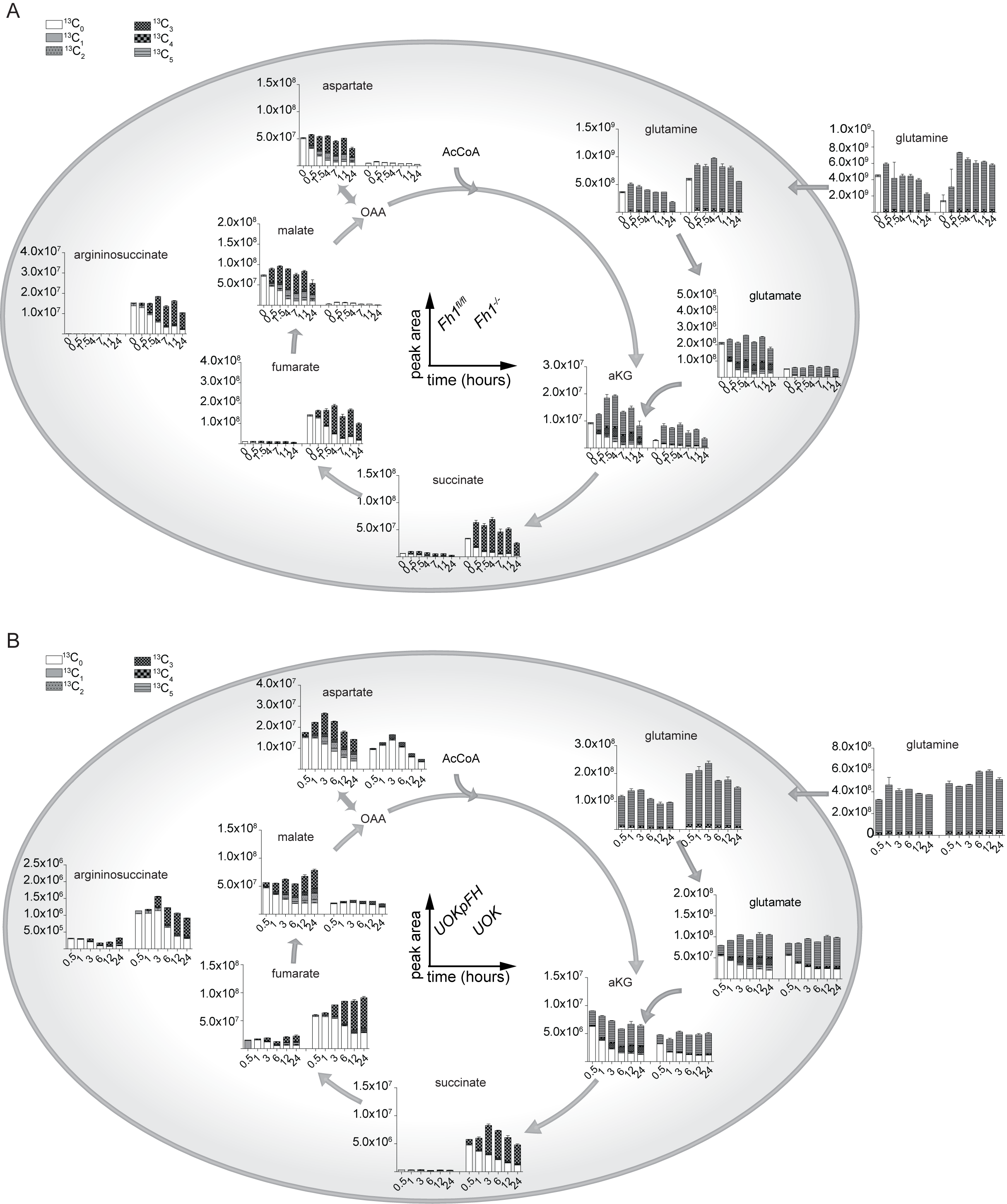

Supplement: Additional file 3 — Isotopologue distribution of TCA cycle metabolites after incubation with U- 13 C-glutamine. (A) Mouse and (B) human FH-deficient or proficient cell lines were incubated for the indicated time with 2 mmol/L U-13C-glutamine, and both the spent media and the intracellular metabolites were analyzed by LC-MS. The isotopologue composition is indicated in the legend and presented as peak area. FH, fumarate hydratase; LC-MS, liquid chromatography-mass spectrometry; TCA, tricarboxylic acid. [file 2049-3002-1-12-S3.tiff]

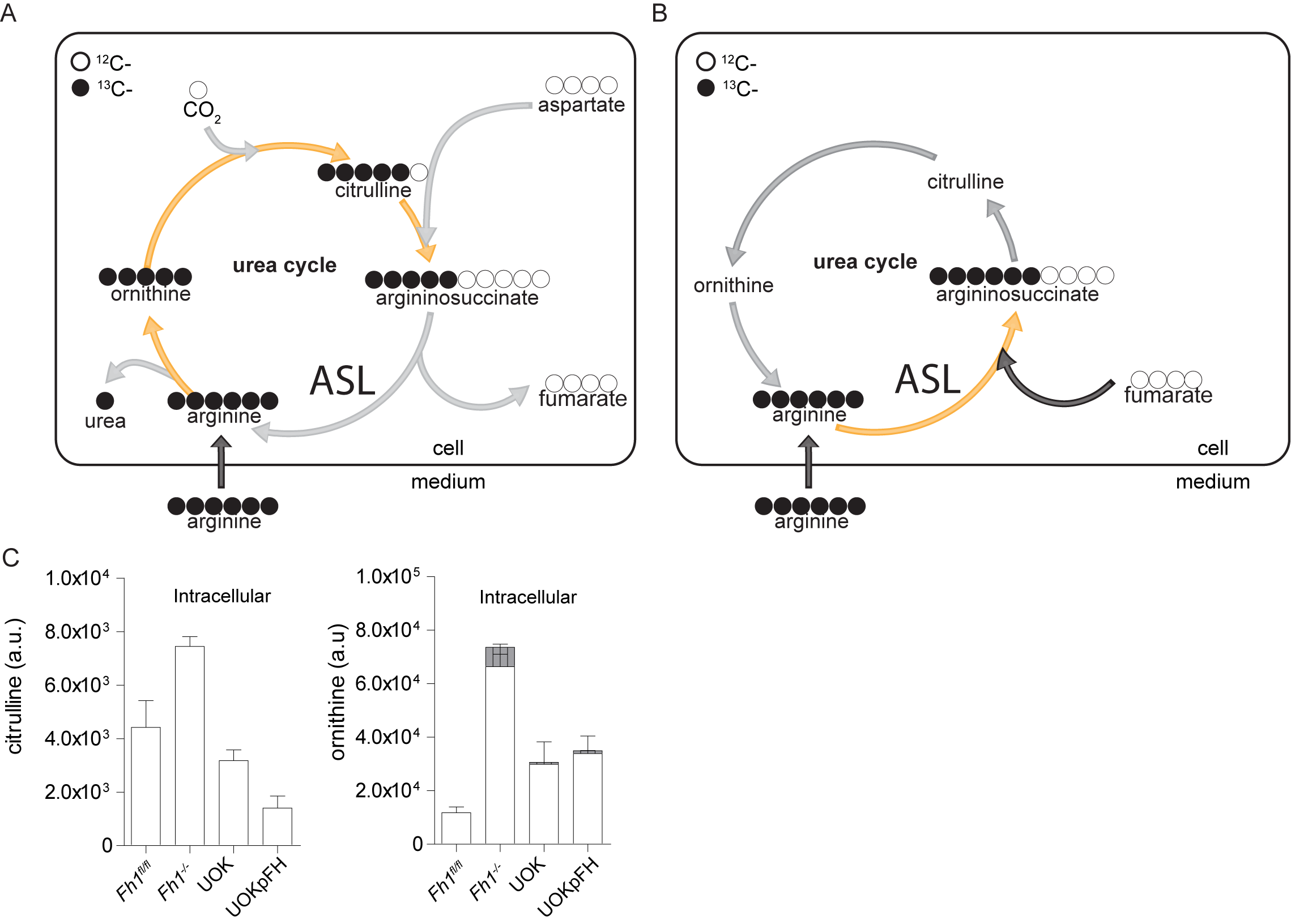

Supplement: Additional file 4 — Schematic representation of the hypothetic labeling profiles of argininosuccinate from U- 13 C-arginine-labelling experiments. After the incubation with labeled arginine, argininosuccinate can be labeled either as (A) 13C5 due to a fully functional urea cycle, or (B) as 13C6 if arginine is converted directly into argininosuccinate by the reversed activity of ASL. (C) Citrulline and ornithine detection by LC-MS in cells incubated with U-13C-arginine. ASL, argininosuccinate lyase; LC-MS, liquid chromatography-mass spectrometry. [file 2049-3002-1-12-S4.tiff]
